# Invasive pneumococcal disease in persons with predisposing factors is dominated by non-vaccine serotypes in Southwest Sweden

**DOI:** 10.1186/s12879-021-06430-y

**Published:** 2021-08-04

**Authors:** Karin Bergman, Tor Härnqvist, Erik Backhaus, Birger Trollfors, Mats S. Dahl, Helena Kolberg, Gunilla Ockborn, Rune Andersson, Johanna Karlsson, Åsa Mellgren, Susann Skovbjerg

**Affiliations:** 1 Department of Infectious Diseases, Institute of Biomedicine, Sahlgrenska Academy, University of Gothenburg, Gothenburg, Sweden; 2 Department of Infectious Diseases, South Älvsborg Hospital, SE-501 82 Borås, Region Västra Götaland Sweden; 3 Department of Infectious Diseases, North Älvsborg Uddevalla Hospital Group, Trollhättan, Region Västra Götaland Sweden; 4 Department of Infectious Diseases, Skaraborg Hospital, Skövde, Region Västra Götaland Sweden; 5 Queen Silvia Children’s Hospital, Sahlgrenska University Hospital, Gothenburg, Region Västra Götaland Sweden; 6 Närhälsan Management Group, Gothenburg, Region Västra Götaland Sweden; 7 Department of Communicable Disease Control, Region Västra Götaland Borås, Sweden; 8 Department of Clinical Microbiology, Sahlgrenska University Hospital, Gothenburg, Region Västra Götaland Sweden; 9 Department of Infectious Diseases, Sahlgrenska University Hospital, Gothenburg, Region Västra Götaland Sweden

**Keywords:** Invasive pneumococcal disease, Pneumococcal infections, Pneumococcal serotype, Pneumococcal conjugated vaccine, PCV13

## Abstract

**Background:**

The pneumococcal conjugate vaccine PCV7 was introduced in Southwest Sweden in the child vaccination program in 2009, followed by PCV13 in 2010 and PCV10 in 2015. In this retrospective cohort study we assessed the pneumococcal serotype distribution in relation to predisposing factors, clinical manifestations and outcome during seven years after PCV introduction.

**Methods:**

Clinical data from 1278 patients with 1304 episodes of invasive pneumococcal disease (IPD) between January 2009 and December 2015 in Region Västra Götaland, Sweden, were retrospectively collected from medical records. Pneumococcal isolates were serotyped by gel diffusion and/or Quellung reactions performed at the Public Health Agency in Sweden. Associations between serotypes and clinical characteristics were statistically evaluated by use of Fisher’s exact test, Mann-Whitney U test and Logistic regression analysis, whereas IPD episodes caused by serotypes over time were analyzed by Mantel-Haenszel chi-square test.

**Results:**

With the exception of serotype 3, the prevalence of PCV13 serotypes decreased during the study period, from 76% (*n* = 157) of all IPD episodes in 2009 to 25% (*n* = 42) in 2015 (*p* < 0.001) while non-PCV13 serotypes increased, mainly among patients ≥65 years and in patients with predisposing factors, including cardiovascular disease, pulmonary disease and malignancy (*p* < 0.001 for all). Patients with predisposing factors, including those with malignancy, immune deficiency or renal disease, were more likely to have IPD caused by a serotype not included in PCV13 rather than a vaccine-included serotype. Serotype 3 was associated with intensive care unit admissions while serotype 1 and 7F caused IPD among healthier and younger patients. PCV13 serotypes were associated with invasive pneumonia, and non-PCV13 serotypes were associated with bacteremia with unknown focus and with manifestations other than pneumonia or meningitis.

**Conclusions:**

Non-PCV13 serotypes caused the majority of IPD cases in Southwest Sweden, especially in patients ≥65 years and in patients with predisposing factors. Serotype 3, included in PCV13, was prevalent and often caused severe disease.

**Supplementary Information:**

The online version contains supplementary material available at 10.1186/s12879-021-06430-y.

## Introduction

Invasive pneumococcal disease (IPD) caused by the bacterium *Streptococcus pneumoniae* remains a major cause of morbidity and mortality despite the introduction of pneumococcal conjugate vaccines (PCVs) in the child vaccination programs worldwide [1].

In January 2009, the conjugate vaccine PCV7 covering serotypes 4, 6B, 9 V, 14, 18C, 19F and 23F was offered to all children in Sweden at three, five and 12 months of age with no catch-up campaign for older children. PCV7 was, a year later replaced by the 10- or 13-valent conjugate vaccines (PCV10, PCV13) with the addition of serotypes 1, 5 and 7F (PCV10) and further with 3, 6A and 19A (PCV13). PCV13 was approved in Sweden for adults in 2011 and has, since 2016 been recommended for adults with specified predisposing factors, in addition to the polysaccharide pneumococcal vaccine (PPV23) [2].

Following vaccine introduction, there has been a striking reduction of IPD among children below two years of age [3–6]. Furthermore, through herd immunity, the incidence of IPD among unvaccinated adults has declined in many countries, although an increase in non-vaccine serotypes has been observed [7–9]. The latter is attributable to the fact that non-vaccine serotypes have replaced vaccine serotypes as nasopharyngeal colonizers in preschool children [10]. In Sweden around one third to one half of all preschool children were asymptomatic nasopharyngeal carriers of pneumococci in the pre-PCV era. However, there was a shift to non-vaccine serotypes subsequent to PCV introduction [11, 12].

The clinical course of IPD may vary from mild with no sequelae to fulminant septic shock with lethal outcome. In addition to age, factors associated with poor outcome include comorbidities such as hematological malignancies, cardiovascular disease and pulmonary disease [13, 14]. Moreover, some pneumococcal serotypes can be more invasive. They may be associated with particular clinical manifestations or may even be lethal [15–17].

Several studies have assessed the serotype distribution of IPD in the post-vaccine era [3–5, 8, 10, 12, 18] but few related clinical manifestations and outcome to different serotypes [17, 19–22]. The aim of this study was to assess serotype distribution in relation to age, risk factors, clinical manifestations and outcome in patients with IPD seven years after introduction of the PCVs in Southwest Sweden.

## Material and methods

### Patients and study site

All episodes of IPD with known pneumococcal serotype occurring in patients between January 2009 and December 2015 in the Region Västra Götaland, Sweden, were eligible for inclusion in this retrospective cohort study. The region is located in Southwest Sweden and had around 1.6 million inhabitants at the time of the study [23]. PCV7 was introduced for all children in the region in January 2009. This was replaced by PCV13 in January/February 2010, and by PCV10 in February 2015.

Patients were included from Sahlgrenska University Hospital, Kungälv Hospital, Southern Älvsborgs County Hospitals, Skaraborg Hospital and the North Älvsborg Uddevalla Hospital Group. Non-permanent residents were excluded. Clinical data regarding age, sex, vaccination status, predisposing factors, manifestations, complications, sequelae, intensive care unit (ICU) admission and case-fatality rate (CFR; death within 30 days of positive culture) were retrospectively collected from medical records using the same protocol as for previous pre-vaccine studies [13, 14, 24]. Predisposing factors included current and previous smoking, cardiovascular disease, malignancy, pulmonary disease, diabetes mellitus, immunosuppressive treatment, substance abuse, autoimmunity, liver disease, immune deficiency, renal disease and asplenia. A second review of the patient records was performed if the collected data were inconclusive. If the same patient had another IPD episode after 30 days or more, then these two were considered as two separate episodes.

### IPD and clinical manifestations

An episode of IPD was defined as a positive culture of pneumococci from a normally sterile site, i.e. blood, cerebrospinal fluid (CSF), pleural fluid or synovial fluid. Clinical manifestations were recorded and defined as follows: Meningitis was diagnosed by growth of pneumococci in culture from the CSF or positive blood culture in combination with detection of pneumococcal DNA by PCR in CSF. Pneumonia was diagnosed by typical appearance on a chest x-ray and/or clinical symptoms compatible with a pneumonia diagnosis in combination with pneumococcal growth in blood culture. A blood culture positive for pneumococci without focal symptoms was considered as bacteremia with unknown focus. Manifestations other than meningitis or pneumonia included abscess, appendicitis, bronchitis, cholangitis, endocarditis, epidural abscess, epiglottitis, mastoiditis, osteitis, otitis media, pharyngitis, peritonitis, prosthetic joint infection, septic arthritis, skin and soft tissue infection, sinusitis and uteritis, and were diagnosed using standard diagnostic methods. These episodes were included only if there was simultaneous growth of pneumococci in blood culture, or in culture from any other normally sterile site.

### *S. pneumoniae* isolation and serotyping

*S. pneumoniae* was cultured and identified using standard microbiological methods at any of the four clinical microbiological laboratories serving the region. Molecular detection of pneumococci in CSF by PCR was performed at Sahlgrenska University Hospital using a previously described method [25]. Serotype identification was performed at the Public Health Agency of Sweden using gel diffusion and/or Quellung reactions [26].

One patient was found to have two different pneumococcal strains in the blood culture; one strain identified as serotype 7F while the other one was non-typeable. In this case, only 7F was included in the analysis.

The study was approved by the Regional Ethics Committee of Gothenburg (no 123–15 and T351–16).

### Statistical calculations

Categories are presented as numbers, and percentages, with ages with median. Differences in clinical characteristics for a specific serotype versus all other serotypes were tested by Fisher’s exact test. Cases including both meningitis and pneumonia were analyzed for both manifestations. Mann-Whitney U test was used to analyze associations between serotypes and age. IPD episodes caused by PCV10, PCV13, PPV23 and non-PCV13 serotypes were analyzed over time (by year) for total number of cases using the Mantel-Haenszel chi-square test. Furthermore, this test was also used to analyze PCV13 and non-PCV13 serotypes over time (by year) for a) the three age groups which were subdivided as follows: < 5, 5–64 and ≥ 65 years; and b) predisposing factors including malignancy and/or pulmonary or cardiovascular disease. Logistic regression was used for evaluation of the background variables and predisposing factors for each serotype and non-vaccine serotypes versus all serotypes, and the results were presented as univariable and multivariable odds ratios (ORs) with 95% confidence interval (CI) and *p*-values. Similarly, non-PCV10 serotypes, non-PCV13 and non-PPV23 serotypes were compared with all serotypes, respectively.

Statistical tests were performed as two-sided with alpha set at 0.05. The analyses were performed using SPSS 25, 26 and 27 Statistics (IBM Corp., Armonk, NY, USA) and SAS 9.4 (SAS Institute Inc., Cary, NC, USA).

## Results

### Study population

**Fig. 1:**
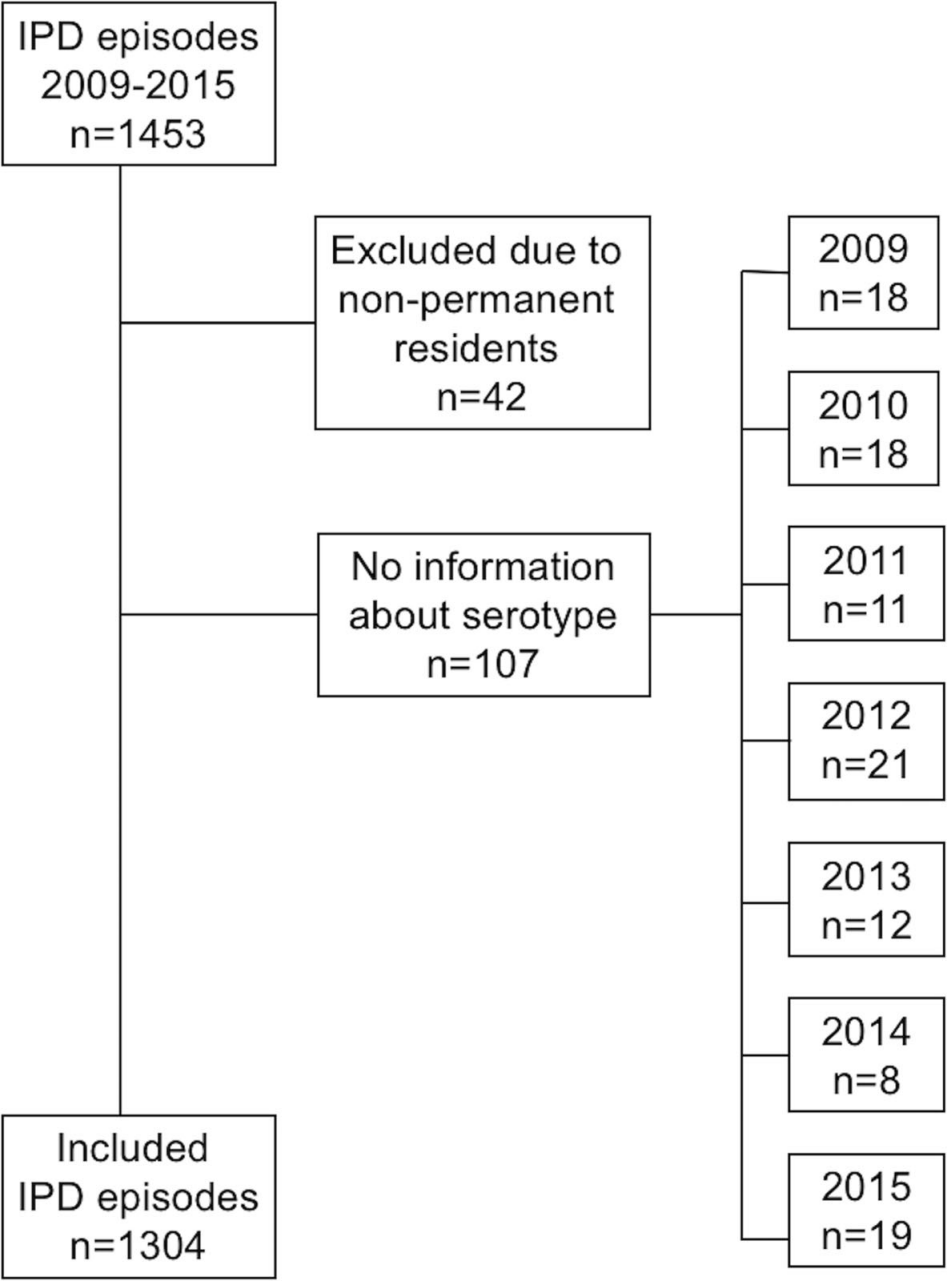
Included episodes of invasive pneumococcal disease (IPD) in Southwest Sweden from 2009 to 2015

Out of 1453 IPD episodes identified between January 2009 and December 2015, serotype results were available for 1346 of these episodes (Fig. 1). Forty-two episodes occurred in non-permanent residents in the area and were therefore excluded. The remaining 1304 (90%) IPD episodes in 1278 patients were analyzed. One patient had three IPD episodes, 20 patients had two, and the remaining patients had one episode. The number of IPD episodes varied from a maximum of 216 episodes in 2010, to a minimum of 154 in 2014. The median age of the patients was 69 years, with an equal distribution of women and men (49 and 51%). Sixty-seven episodes (5%) occurred in patients who were vaccinated with either PCV7, PCV10, PCV13 and/or PPV23. Almost half of these patients (*n* = 31) were infected by a serotype included in the vaccine which they had received. However, the timepoint for the vaccination was not registered. No child below the age of five who had received all doses of the vaccine according to the child vaccination program, had IPD with a pneumococcal serotype included in the current vaccine.

### Changes in serotype distribution

**Fig. 2:**
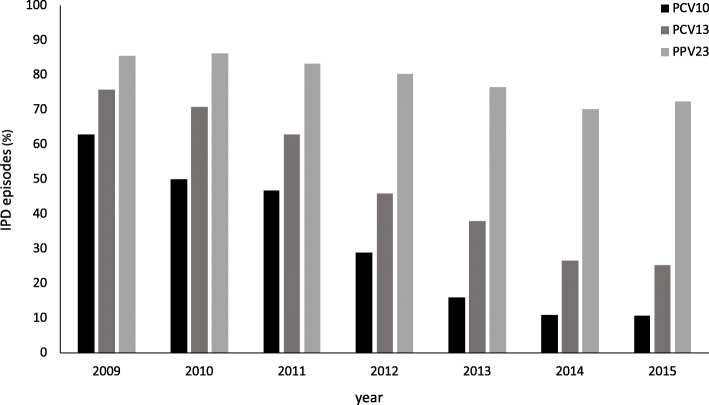
The proportion of vaccine-included serotypes causing invasive pneumococcal disease (IPD) in patients in Southwest Sweden between 2009 and 2015. Number of episodes in 2009 *n* = 207, 2010 *n* = 216, 2011 *n* = 197, 2012 *n* = 177, 2013 *n* = 187, 2014 *n* = 154, 2015 *n* = 166. PCV10 = 10-valent pneumococcal conjugate vaccine, PCV13 = 13-valent pneumococcal conjugate vaccine, PPV23 = 23-valent pneumococcal polysaccharide vaccine

**Fig. 3:**
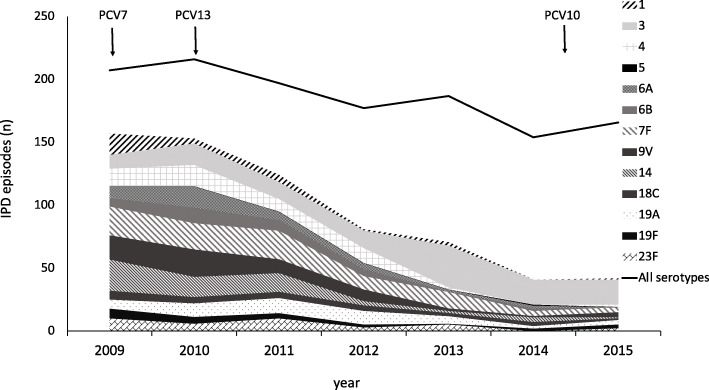
The distribution of the serotypes included in the 13-valent pneumococcal conjugate vaccine (PCV13) in patients with invasive pneumococcal disease (IPD) between 2009 and 2015 in Southwest Sweden. The arrows indicate the introduction of PCV7 and the shift to PCV13 and PCV10 in the child vaccination program. PCV7 = 7-valent pneumococcal conjugate vaccine, PCV10 = 10-valent pneumococcal conjugate vaccine, PCV13 = 13-valent pneumococcal conjugate vaccine

**Fig. 4:**
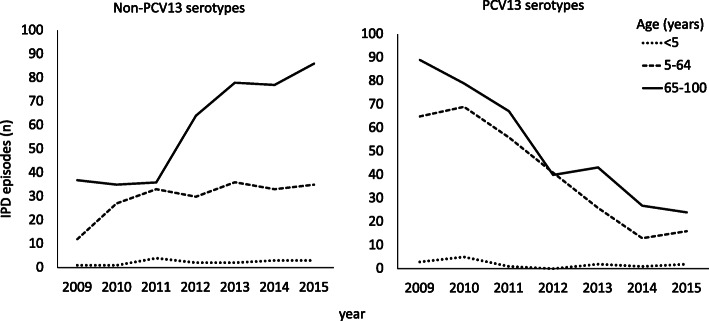
Non-PCV13 and PCV13 serotypes causing invasive pneumococcal disease (IPD) in the age groups < 5 years (*n* = 30), 5–64 years (*n* = 492) and 65–100 years (*n* = 782) in Southwest Sweden between 2009 and 2015. PCV13 = 13-valent pneumococcal conjugate vaccine

A total of 45 different serotypes were identified in the IPD cases during the study period, the most common of these being 22F and 3, both causing 10% of the IPD episodes, followed by 7F, causing 8% of the cases (Table 1). With the exception of serotype 3, the PCV13 serotypes diminished gradually during the observation period, causing 76% (*n* = 157) of all IPD episodes in 2009 and 25% (*n* = 42) in 2015 (*p* < 0.001) (Figs. 2 and 3). Likewise, the serotypes included in PCV10 declined from 63% in 2009 to 11% in 2015 (*p* < 0.001) and for PPV23 from 86 to 72%, (*p* < 0.001) (Fig. 2). Contrary to this, non-PCV13 serotypes increased during this period (*p <* 0.001). The most prevalent non-PCV13 serotype, 22F, increased from 5% (*n* = 10) in 2009 to 16% (*n* = 27) in 2015. The increase in non-PCV13-related IPD was most pronounced in the age group ≥65 years (Fig. 4). Among young children (< 5 years), no shift in serotype distribution was reported although low numbers of cases of IPD among young children were found (*n* = 30).

**Table 1:** The distribution of serotypes and clinical characteristics in patients with invasive pneumococcal disease (IPD) caused by the 24 most prevalent serotypes in Southwest Sweden between 2009 and 2015

		Clinical characteristics							
Serotype	Number of strains, n (%)	Median age (y)	Predisposing factors^**a**^, n (%)	Meningitis#, n (%)	Pneumonia#, n (%)	Bacteremia with unknown focus, n (%)	Other manifestation^**b**^, n (%)	Admitted to the intensive care unit, n (%)	Case fatality rate^**c**^, n (%)
**22F**	135 (10)	70	112 (83)	10 (7)	92 (68)	17 (13)	16 (12)*	30 (22)	17 (13)
**3**	128 (10)	68	99 (77)	15 (12)	100 (78)	8 (6)*	5 (4)	46 (36)***	23 (18)
**7F**	99 (8)	60***	65 (66)***	4 (4)	84 (85)**	5 (5)**	7 (7)	17 (17)	4 (4)**
**9 V**	68 (5)	65	54 (79)	2 (3)	59 (87)**	4 (6)	3 (4)	16 (24)	4 (6)
**14**	67 (5)	75	53 (79)	4 (6)	60 (90)***	2 (3)**	1 (2)	16 (24)	9 (13)
**33F**	59 (5)	67	49 (83)	7 (12)	35 (59)*	13 (22)*	4 (7)	12 (20)	5 (8)
**4**	57 (4)	64	47 (83)	2 (4)	49 (86)*	4 (7)	3 (5)	20 (36)*	4 (7)
**19A**	53 (4)	75*	43 (81)	3 (6)	39 (74)	9 (17)	2 (4)	16 (30)	10 (19)
**11A**	49 (4)	70	45 (92)*	4 (8)	35 (71)	7 (14)	4 (8)	14 (29)	10 (20)
**9 N**	47 (4)	71	42 (89)	1 (2)	38 (81)	5 (11)	4 (9)	7 (15)	4 (9)
**8**	43 (3)	66	35 (81)	2 (5)	34 (79)	4 (9)	3 (7)	4 (9)*	2 (5)
**6A**	42 (3)	74	33 (79)	2 (5)	29 (69)	9 (21)	2 (5)	11 (26)	6 (14)
**23F**	36 (3)	70	29 (81)	1 (3)	23 (64)	5 (14)	6 (17)*	4 (11)	2 (6)
**23A**	35 (3)	75	30 (86)	5 (14)	20 (57)	6 (17)	4 (11)	6 (17)	5 (14)
**6B**	34 (3)	75	29 (85)	4 (12)	27 (79)	3 (9)	0 (0)	6 (18)	7 (21)
**1**	32 (2)	49***	18 (56)**	1 (3)	30 (94)**	1 (3)	0 (0)	2 (6)*	1 (3)
**18C**	27 (2)	66	20 (74)	5 (19)	17 (63)	3 (11)	2 (7)	6 (22)	2 (7)
**35F**	26 (2)	73	24 (92)	1 (4)	18 (69)	7 (27)	0 (0)	4 (15)	4 (15)
**23B**	26 (2)	75	19 (73)	4 (15)	8 (31)***	10 (39)**	4 (15)	9 (35)	3 (12)
**6C**	25 (2)	82**	20 (80)	0 (0)	20 (80)	4 (16)	1 (4)	5 (20)	4 (16)
**19F**	25 (2)	68	20 (80)	3 (12)	16 (64)	5 (20)	1 (4)	7 (28)	5 (20)
**12F**	24 (2)	70	17 (71)	3 (13)	14 (58)	3 (13)	4 (17)	6 (25)	3 (13)
**10A**	24 (2)	72	23 (96)	3 (13)	12 (50)*	6 (25)	3 (13)	6 (25)	6 (25)
**15C**	20 (2)	68	15 (75)	5 (25)*	8 (40)**	7 (35)*	0 (0)	5 (25)	3 (15)
**Other** ^**d**^	123 (9)	71	110 (89)	12 (10)	76 (62)	26 (21)	11 (9)	30 (24)	22 (18)
**Total**	1304	69	1051 (81)	102 (8)	943 (72)	173 (13)	90 (7)	302(23)	165 (13)

* *p* < 0.05, ** *p* < 0.01, *** *p* < 0.001; versus total

# There were five episodes of both pneumonia and meningitis. These were included in the analysis of each of the manifestations

^a^ Smoking, cardiovascular disease, malignancy, pulmonary disease, diabetes mellitus, immunosuppressive treatment, substance abuse, autoimmunity, liver disease, immune deficiency, renal disease and asplenia

^b^Abscess, appendicitis, bronchitis, cholangitis, endocarditis, epidural abscess, epiglottitis, mastoiditis, osteitis, otitis media, pharyngitis, peritonitis, prosthetic joint infection, septic arthritis, skin and soft tissue infection, sinusitis and uteritis

^c^Death within 30 days of positive culture for pneumococci

^d^Including serotype 15B (n = 19), 35B (n = 17), 16F (n = 13), 31 (*n* = 11), 15A (n = 11), 38 (n = 10), 24F (n = 9), 20 (n = 7), 17F (n = 6), 10B (n = 3), 34 (n = 3), Not typable (n = 3), 18A (n = 2), 7C (n = 2), 35A (n = 1), 9A (n = 1), 23 (*n* = 1), 9 (*n* = 1), 21 (n = 1), 11 (n = 1) and 5 (n = 1)

### Changes in serotype distribution in relation to predisposing factors

**Fig. 5:**
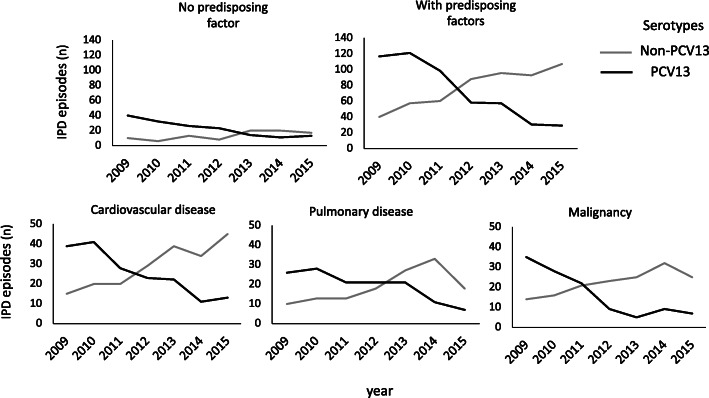
PCV13 and non-PCV13 serotypes causing invasive pneumococcal disease (IPD) in patients with or without predisposing factors (*n* = 1051 and *n* = 253, respectively), including cardiovascular disease (*n* = 379), malignancies ((*n* = 271), including solid tumors (*n* = 149) and hematologic malignancies (*n* = 131)), and pulmonary disease (*n* = 267) between the years 2009 and 2015. PCV13 = 13-valent pneumococcal conjugate vaccine

The vast majority, 1051 (81%), of all episodes occurred in patients having one or more predisposing factors, the most prevalent of these being cardiovascular disease (*n* = 379, 36%), malignancy (*n* = 271, 26%) and pulmonary disease (*n* = 267, 25%). The shift in serotype distribution was modest in episodes occurring in patients without any known risk factors. However, this shift was much more pronounced in episodes occurring in patients with predisposing factors (Fig. 5). In 2015, non-PCV13 serotypes caused 79% (107/136) of the IPD episodes in patients with any predisposing factors. This had increased from 25% (40/157) in 2009 (*p* < 0.001). Similar patterns were observed for patients with cardiovascular disease, malignancy and pulmonary disease (*p* < 0.001 for all) (Fig. 5).

### Associations between predisposing factors and serotypes

Both non-PCV10 and non-PCV13 serotypes were significantly associated with IPD in patients having any predisposing factor, as compared with the serotypes included in these vaccines (Tables 2 and 3). Logistic regression analysis further revealed that current malignancy or an immune deficiency was positively associated with non-PCV13 and non-PPV23 serotypes (Table 3). This was also the case for renal disease and non-PCV13 serotypes (Table 3). In contrast, no positive association with any predisposing factor was shown for serotype 3 (Additional Table 1). The median age was significantly lower in IPD cases caused by serotype 1 and 7F (both included in the PCV13). Having a predisposing factor was less common in patients having IPD with these two serotypes (Table 1).

**Table 2:** The distribution of vaccine and non-vaccine pneumococcal serotypes in relation to clinical factors in patients with invasive pneumococcal disease in Southwest Sweden between 2009 and 2015

		Clinical characteristics							
Serotype	Number of strains n (%)	Median age (y)	Predisposing factors^a^ n (%)	Meningitis# n (%)	Pneumonia# n (%)	Bacteremia with unknown focus n (%)	Other manifestation^**b**^ n (%)	Admitted to the intensive care unit n (%)	Case fatality rate^**c**^ n (%)
**PCV10**^**d**^	446 (34)	65	335 (75)***	26 (6)	366 (82)***	32 (7)***	23 (5)	94 (21)	38 (9)**
**Non-PCV10**	858 (66)	71	716 (83)	76 (9)	577 (67)	141 (16)	67 (8)	208 (24)	127 (15)
**PCV13**^**e**^	669 (51)	67	510 (76)***	46 (7)	534 (80)***	58 (9)***	32 (5)**	167 (25)	77 (12)
**Non-PCV13**	635 (49)	71	541 (85)	56 (9)	409 (64)	115 (18)	58 (9)	135 (21)	88 (14)
**PPV23**^**f**^	1040 (80)	68	829 (80)	77 (7)	788 (76)***	110 (11)***	69 (7)	241 (23)	124 (12)
**Non-PPV23**	264 (20)	74	222 (84)	25 (9)	155 (59)	63 (24)	21 (8)	61 (23)	41 (16)
**Total**	1304	69	1051 (81)	102 (8)	943 (72)	173 (13)	90 (7)	302 (23)	165 (13)

The statistical calculations were performed by comparing PCV10 serotypes versus non-PCV10 serotypes, PCV13 serotypes versus non-PCV13 serotypes and PPV23 serotypes versus non-PPV23 serotypes, respectively

* *p* < 0.05, ** *p* < 0.01, *** *p* < 0.001

# There were five episodes of both pneumonia and meningitis. These were included in the analysis of each of the manifestations

^a^ Smoking, cardiovascular disease, malignancy, pulmonary disease, diabetes mellitus, immunosuppressive treatment, substance abuse, autoimmunity, liver disease, immune deficiency, renal disease and asplenia

^b^Abscess, appendicitis, bronchitis, cholangitis, endocarditis, epidural abscess, epiglottitis, mastoiditis, osteitis, otitis media, pharyngitis, peritonitis, prosthetic joint infection, septic arthritis, skin and soft tissue infection, sinusitis and uteritis

^c^Death within 30 days of positive culture for pneumococci

^d^Including serotype 1, 4, 5, 6B, 7F, 9 V, 14, 18C, 19F and 23F

^e^Including serotype 1, 3, 4, 5, 6A, 6B, 7F, 9 V, 14, 18C, 19A, 19F and 23F

^f^Including serotype 1, 2, 3, 4, 5, 6B, 7F, 8, 9 N, 9 V, 10A, 11A, 12F, 14, 15B, 17F, 18C, 19F, 19A, 20, 22F, 23F and 33F

**Table 3:** Univariable and multivariable odds ratios (OR) with 95% confidence intervals (CI) for invasive pneumococcal disease with non-vaccine serotypes in relation to patient derived factors in Southwest Sweden between 2009 and 2015

	Odds ratio (OR) with 95% confidence interval (CI)											
	Univariable analysis						Multivariable analysis					
**Variable**	**Non-PCV10 serotypes,** ***n*** **= 858**	***p***	**Non-PCV13 serotypes,** ***n*** **= 635**	***p***	**Non-PPV23 serotypes,** ***n*** **= 264**	***p***	**Non-PCV10 serotypes, n = 858**	***p***	**Non-PCV13 serotypes,** ***n*** **= 635**	***p***	**Non-PPV23 serotypes,** ***n*** **= 264**	***p***
**Age (by 10 years)**	**1.12 (1.06–1.19)**	**< 0.0001**	**1.09 (1.03–1.15)**	**0.002**	**1.12 (1.04–1.20)**	**0.002**	**1.01 (1.00–1.02)**	**0.001**			**1.01 (1.01–1.02)**	**0.006**
**Men,** ***n*** **= 668**	0.96 (0.76–1.21)	0.77	0.98 (0.79–1.22)	0.89	1.05 (0.81–1.39)	0.70						
**Predisposing factor, any, n = 1051**	**1.66 (1.26–2.22)**	**0.0003**	**1.79 (1.35–2.38)**	**< 0.0001**	1.35 (0.93–1.92)	0.11	**1.47 (1.10–1.96)**	**0.01**	**1.52 (1.14–2.04)**	**0.005**		
**Current or previous smoking,** ***n*** **= 528**	0.91 (0.72–1.14)	0.45	1.02 (0.82–1.28)	0.83	**0.77 (0.58–1.02)**	**0.07**						
**Cardiovascular disease, n = 379**	**1.56 (1.19–2.00)**	**0.001**	**1.30 (1.02–1.64)**	**0.03**	**1.32 (0.99–1.75)**	**0.06**						
**Malignancy, n = 271**	**1.36 (1.02–1.81)**	**0.04**	**1.56 (1.19–2.04)**	**0.001**	**1.79 (1.32–2.44)**	**0.0002**			**1.37 (1.04–1.82)**	**0.03**	**1.61 (1.18–2.22)**	**0.003**
**Pulmonary disease, n = 267**	1.19 (0.89–1.58)	0.23	1.04 (0.79–1.35)	0.79	1.09 (0.78–1.52)	0.62						
**Diabetes mellitus,** ***n*** **= 195**	1.04 (0.75–1.44)	0.78	1.08 (0.79–1.45)	0.64	0.94 (0.65–1.39)	0.78						
**Immunsuppressive treatment,** ***n*** **= 170**	1.06 (0.75–1.49)	0.71	1.18 (0.85–1.64)	0.31	1.30 (0.88–1.89)	0.18						
**Substance abuse,** ***n*** **= 116**	0.86 (0.58–1.29)	0.50	0.78 (0.53–1.15)	0.21	0.65 (0.38–1.11)	0.12						
**Autoimmunity,** ***n*** **= 106**	0.88 (0.58–1.33)	0.56	1.02 (0.68–1.52)	0.94	1.32 (0.83–2.08)	0.25						
**Liver disease,** ***n*** **= 64**	1.47 (0.82–2.56)	0.19	1.12 (0.68–1.85)	0.64	1.33 (0.75–2.38)	0.33						
**Immune deficiency, n = 63**	**1.85 (1.02–3.44)**	**0.04**	**2.56 (1.47–4.35)**	**0.0009**	**2.22 (1.30–3.85)**	**0.003**			**2.17 (1.23–3.70)**	**0.007**	**2.13 (1.23–3.70)**	**0.007**
**Renal disease, n = 63**	**1.85 (1.02–3.44)**	**0.04**	**2.17 (1.28–3.70)**	**0.004**	1.37 (0.76–2.44)	0.30			**1.96 (1.15–3.33)**	**0.01**		

The statistical calculations were performed by comparing non-PCV10 serotypes versus all serotypes, non-PCV13 serotypes versus all serotypes and non-PPV23 serotypes versus all serotypes, respectively

### Clinical manifestations

IPD with pneumonia occurred in 72% (*n* = 943) of episodes. In this group, serotype 3 was the most prevalent (*n* = 100) followed by 22F (*n* = 92) and 7F (*n* = 84) (Table 1). However, serotypes significantly associated with invasive pneumonia included 14, 7F, 9 V, 1 and 4 (Table 1). An overall association between PCV13 and PCV10 serotypes, respectively, and invasive pneumonia, as compared to the non-PCV serotypes was also observed (*p* < 0.001 for both) (Table 2). In total, 102 IPD episodes (8%) were associated with meningitis, including 30 different pneumococcal serotypes. The most prevalent was serotype 3, followed by serotype 22F and 33F (Table 1). A significant association was found between serotype 15C and meningitis (*p =* 0.016). PCV13 serotypes were equally as common as non-PCV13 serotypes (Table 2).

Bacteremia with unknown focus was most often caused by serotype 22F (*n* = 17) or 33F (*n* = 13) while serotype 23B was significantly associated with the condition (Table 1). The non-PCV13 serotypes more frequently caused IPD with unknown focus, as well as IPD with other manifestations including otitis media, septic arthritis, sinusitis, endocarditis, and spondylodiscitis rather than the PCV13 serotypes (*p* < 0.001 and *p* = 0.002, respectively) (Table 2).

### Admission to the intensive care unit and case fatality rate

IPD caused by serotype 3 was a predictor of ICU admissions, and 36% (46/128) of the IPD episodes caused by serotype 3 demanded ICU-care as compared to 23% of all episodes (*p =* 0.0006) (Table 1). Serotype 4 was also associated with ICU-care with admissions at 36% of cases (*p =* 0.034). The overall case fatality rate (CFR) was 13% and non-PCV10 serotypes were associated with a higher CFR compared to PCV10 serotypes (15 vs 9%, *p* = 0.001) (Table 2) although no single serotype was associated with an increased risk of death. However, serotype 7F was associated with a higher survival rate (CFR 4%, *p* = 0.004) (Table 1).

## Discussion

Seven years after the introduction of PCV in the child vaccination program the pneumococcal serotypes causing IPD in Southwest Sweden have shifted from a minority of non-PCV13 serotypes in 2009, to the vast majority in 2015, especially among persons ≥65 years of age and, as reported in previous studies, in persons with predisposing factors [21, 22]. Bacteremia with unknown focus and manifestations other than pneumonia and meningitis were more commonly caused by non-PCV13 serotypes than serotypes included in the PCV13. Conclusively, the spectrum of serotypes as well as the clinical manifestations of IPD have changed subsequent to the PCV introduction into the child vaccination program and the resulting herd immunity.

The overall case fatality rate (CFR) was 13% in our study; however, no specific serotype was significantly associated with a higher CFR. Instead, a lower CFR was found among patients having IPD caused by serotype 7F. Serotype 3 has previously been associated with lethal outcome in patients with IPD, septic shock, and also bacteremic pneumonia [19, 22, 27, 28]. In this study the CFR was 18% for serotype 3, supporting previous observations that IPD caused by serotype 3 is severe. We further found that patients with IPD caused by serotypes 3 and 4 required ICU-care to a greater extent than IPD caused by other serotypes. Other non-bacterial factors, including predisposing factors and age may result in admissions to ICU-care. However, we found no association between serotype 3 and any predisposing factor or advanced age.

The capsule of serotype 3 is thick due to an extended polysaccharide layer which favors nasopharyngeal carriage [27]. It is therefore less likely to be invasive compared to other serotypes. However, if it does invade, it is more prone to cause severe disease [27]. Most, but not all, studies show a poor protection of PCV13 against serotype 3 [8, 18, 29, 30]. Here we show that serotype 3 is still prevalent, despite an overall decrease in PCV13 serotypes. In fact, serotype 3 was the most prevalent serotype in IPD cases in mainly the same area 30–40 years ago [16], subsequently losing its top position before the introduction of PCVs in favor of serotypes 1, 7F and 9 V [15], and then regaining a top position together with serotype 22F post-PCV, as shown here. Serotype 3 was also the most prevalent serotype in Stockholm, Sweden, after the vaccine introduction, mainly due to the widespread CC180 clone among the elderly [12]. Thus, there are temporal trends in serotypes circulating in the population at the time [21, 31] in addition to vaccine effects.

The changes in serotype distribution found here are in concordance with studies from US and Western Europe. These also show a non-PCV serotype dominance among IPD cases, with serotype 3 being the most prevalent in many countries [4, 8, 21, 22, 32].

There are pneumococcal serotypes with more invasive potential, including 1, 5 and 7F, which are infrequent colonizers of the nasopharynx [33]. In parallel with previous studies [15, 34], we found that serotypes 1 or 7F were associated with invasive pneumonia, low CFR (3–4%) and more often occurred in young and otherwise healthy individuals. The most common clone of serotype 1 (MLST306) expresses a non-hemolytic pneumolysin that aids the bacteria in avoiding the innate immune system. It is believed that this increases invasiveness though it is less fatal [35]. During the study period, probably through vaccine-effect and herd immunity, both serotypes became scarcer.

As shown in the multivariable analysis, having one or more predisposing factors was associated with having IPD caused by a non-PCV10 or a non-PCV13 serotype. More specifically, malignancy and immune deficiency were both positively associated with IPD caused by non-PCV13 and non-PPV23 serotypes. We observed these positive associations only between the various predisposing factors and IPD caused by non-vaccine serotypes. This demonstrates that serotypes not included in the current vaccines are overall more prone to cause disease among persons with predisposing factors than are those serotypes included in the vaccines.

The non-PCV13 serotypes constituted 75% of all IPD cases in 2015 and the trend remained the same in 2020, on a national level [36]. Among persons with predisposing factors, the non-PCV13 serotypes clearly dominated. This was similar to the US and UK in the post-vaccine era [21, 22, 32]. This raises the question as to the rationale for recommendations of PCV13 for adults, including patients with chronic cardiovascular disease, pulmonary disease and malignancies. The Advisory Committee on Immunization Practices and the Public Health Agency in Sweden recently revised their recommendations for PCV13 for immunocompetent adults ≥65 years of age due to the diminishing incidence of PCV13 serotypes [2, 37]. There are ongoing clinical trials being performed with conjugate vaccines covering 15 (PCV13 + 22F and 33F) and 20 (PCV13 + 8, 10A, 11A, 12F, 15 BC, 22F and 33F) pneumococcal serotypes [38, 39]. Based on the data presented in this study, these vaccines would have covered 46% (77/166) and 64% (107/166), respectively, of the IPD-causing serotypes in Southwest Sweden 2015. They could thus be more appropriate for immunization in adults, in combination with PPV23, which covered 72% of the invasive serotypes in the same year. However, the expectation of a continuous serotype shift indicates the need for continuous surveillance and further vaccine development.

Strengths of this study include the detailed clinical characteristics and outcomes in patients as well as the low incidence of excluded IPD episodes.

Limitations include the use of ICU-care as a proxy for disease severity, and patients with septic shock were not identified. It is possible that information relating to substance abuse and smoking may be limited to the retrospective design of the study. Furthermore, the odds ratios in our univariable and multivariable analyses have to be considered within the context of sample size, and larger-scale studies are warranted to confirm the findings.

## Conclusions

Since the introduction of PCVs into the child vaccination program in Sweden 2009, a serotype shift has occurred with non-PCV13 serotypes becoming most frequent in adults ≥65 years of age and in patients with predisposing factors. This raises the question of whether the use of PCV13, as a supplement to PPV23, should be recommended for adults with predisposing factors.

## Supplementary Information

**Additional file 1: Table S1.** Univarible and multiviariable odds ratios (OR) with 95 % confidence intervals (CI) for invasive pneumococcal disease with specific serotypes. **Table S2.** **Table S3** **Table S4.**

## Data Availability

The datasets used and analyzed during the current study are available from the corresponding author on reasonable request.

## Abbreviations

IPD: Invasive pneumococcal disease
PCV: Pneumococcal conjugate vaccine
PCV7: 7-valent pneumococcal conjugate vaccine
PCV10: 10-valent pneumococcal conjugate vaccine
PCV13: 13-valent pneumococcal conjugate vaccine
PPV23: 23-valent polysaccharide pneumococcal vaccine
ICU: Intensive care unit
CFR: Case fatality rate
CSF: Cerebrospinal fluid
OR: Odds ratio
95% CI: 95% Confidence interval
