# Supplementary material for: Invasive pneumococcal disease in persons with predisposing factors is dominated by non-vaccine serotypes in Southwest Sweden
Source: BMC Infect Dis. 2021 Aug 4;21:756. doi: 10.1186/s12879-021-06430-y (PMC8335464; doi:10.1186/s12879-021-06430-y)
Supplement: Supplementary file 1 — Additional file 1: Table S1. Univarible and multiviariable odds ratios (OR) with 95 % confidence intervals (CI) for invasive pneumococcal disease with specific serotypes. Table S2. Table S3 Table S4. [file 12879_2021_6430_MOESM1_ESM.docx]

**Additional table 1.** Univariable and multivariable odds ratios (OR) with 95% confidence intervals (CI) for invasive pneumococcal disease with PCV13 specific serotype 6A and serotypes 3 and 19A, included in PCV13 and PPV23, in relation to patient derived factors in Southwest Sweden between 2009 and 2015. PCV13= 13-valent pneumococcal conjugate vaccine. PPV23= 23-valent pneumococcal polysaccharide vaccine.

|  | **Odds ratio with 95 % confidence interval** | | | | | | | | | |
| --- | --- | --- | --- | --- | --- | --- | --- | --- | --- | --- |
| **Variable** | **Univariable analysis** | | | | |  | **Multivariable analysis** | | | |
|  | **3** (n=128) | ***p*** | **6A** (n=42) | ***p*** | **19A** (n=53) | ***p*** | **3** (n=128) | ***p*** | **19A** (n=53) | ***p*** |
| **Age (by 10 years)** | 0.96 (0.88-1.05) | 0.41 | 1.11 (0.94-1.32) | 0.22 | **1.21 (1.03-1.43)** | **0.02** |  |  | **1.02 (1.00-1.04)** | **0.02** |
| **Men, n=668** | 0.91 (0.64-1.32) | 0.63 | 1.42 (0.76-2.65) | 0.28 | 0.84 (0.49-1.46) | 0.55 |  |  |  |  |
| **Predisposing factor, any n=1051** | 0.80 (0.52-1.25) | 0.33 | 0.88 (0.42-1.86) | 0.74 | 1.04 (0.51-2.09) | 0.92 |  |  |  |  |
| **Smoking, n=528** | 0.87 (0.60-1.27) | 0.47 | 0.81 (0.43-1.54) | 0.52 | 0.81 (0.46-1.44) | 0.48 |  |  |  |  |
| **Cardiovascular disease, n=379** | 1.22 (0.82-1.80) | 0.33 | 1.10 (0.56-2.13) | 0.78 | 1.27 (0.71-2.27) | 0.42 |  |  |  |  |
| **Pulmonary disease, n=267** | 1.40 (0.92-2.13) | 0.12 | 1.06 (0.50-2.25) | 0.88 | 0.90 (0.45-1.81) | 0.77 |  |  |  |  |
| **Malignancy, n=271** | **0.30 (0.15-0.57)** | **0.0003** | 1.20 (0.58-2.47) | 0.62 | **1.85 (1.02-3.35)** | **0.04** | **0.30 (0.15-0.57)** | **0.0003** |  |  |
| **Diabetes mellitus, n=195** | 0.99 (0.59-1.65) | 0.97 | 1.14 (0.50-2.61) | 0.75 | 0.72 (0.30-1.70) | 0.45 |  |  |  |  |
| **Immunsuppressive treatment, n=170** | **0.48 (0.24-0.96)** | **0.04** | 1.12 (0.46-2.69) | 0.81 | 1.59 (0.78-3.22) | 0.20 |  |  |  |  |
| **Substance Abuse, n=116** | 1.54 (0.88-2.70) | 0.13 | 0.24 (0.03-1.78) | 0.16 | 1.32 (0.55-3.17) | 0.53 |  |  |  |  |
| **Autoimmunity, n=106** | 0.73 (0.35-1.55) | 0.42 | 1.55 (0.60-4.04) | 0.37 | 0.43 (0.10-1.80) | 0.25 |  |  |  |  |
| **Liver disease, n=64** | 1.76 (0.87-3.55) | 0.11 | 0.97 (0.23-4.10) | 0.96 | 0.75 (0.18-3.16) | 0.70 |  |  |  |  |
| **Renal disease, n=63** | 0.29 (0.07-1.20) | 0.09 | 0.47 (0.06-3.49) | 0.46 | 1.65 (0.58-4.72) | 0.35 |  |  |  |  |
| **Immune deficiency, n=63** | 0.14 (0.02-1.03) | 0.05 | 1.54 (0.46-5.13) | 0.48 | 0.37 (0.05-2.71) | 0.33 |  |  |  |  |

**Additional table 2.** Univariable and multivariable odds ratios (OR) with 95% confidence intervals (CI) for invasive pneumococcal disease with serotypes 1, 4 and 6B, included in PCV10, PCV13 and PPV23, in relation to patient derived factors in Southwest Sweden between 2009 and 2015. PCV10= 10-valent pneumococcal conjugate vaccine. PCV13= 13-valent pneumococcal conjugate vaccine. PPV23= 23-valent pneumococcal polysaccharide vaccine. lim(OR)=0= No episodes of invasive pneumococcal disease.

|  | **Odds ratio with 95 % confidence interval** | | | | | | | | | | | |
| --- | --- | --- | --- | --- | --- | --- | --- | --- | --- | --- | --- | --- |
| **Variable** | **Univariable analysis** | | | | |  | **Multivariable analysis** | | | | | |
|  | **1** (n= 32) | ***p*** | **4** (n=57) | ***p*** | **6B** (n=34) | ***p*** | **1** (n= 32) | ***p*** | **4** (n=57) | ***p*** | **6B** (n=34) | ***p*** |
| **Age (by 10 years)** | **0.72 (0.63-0.82)** | **<0.0001** | 0.95 (0.84-1.08) | 0.44 | **1.20 (0.98-1.48)** | **0.08** | **0.97 (0.96-0.98)** | **<0.0001** |  |  |  |  |
| **Men, n=668** | 0.95 (0.47-1.92) | 0.89 | 1.14 (0.67-1.95) | 0.63 | 0.66 (0.33-1.32) | 0.24 |  |  |  |  |  |  |
| **Predisposing factor, any n=1051** | **0.30 (0.15-0.61)** | **0.0009** | 1.14 (0.57-2.28) | 0.72 | 1.41 (0.54-3.67) | 0.48 |  |  |  |  |  |  |
| **Smoking, n=528** | 1.15 (0.57-2.33) | 0.70 | **2.25 (1.31-3.87)** | **0.01** | 1.49 (0.75-2.94) | 0.25 |  |  |  |  |  |  |
| **Cardiovascular disease, n=379** | **0.34 (0.12-0.98)** | **0.05** | 0.95 (0.53-1.72) | 0.87 | 1.34 (0.66-2.74) | 0.42 |  |  |  |  |  |  |
| **Malignancy, n=271** | 0.25 (0.06-1.05) | 0.06 | **0.28 (0.10-0.77)** | **0.01** | 1.61 (0.76-3.41) | 0.21 |  |  | **0.31 (0.11-0.86)** | **0.03** |  |  |
| **Pulmonary disease, n=267** | 0.25 (0.06-1.07) | 0.06 | **2.37 (1.36-4.14)** | **0.01** | 0.66 (0.25-1.73) | 0.40 |  |  | **2.32 (1.32-4.06)** | **0.01** |  |  |
| **Diabetes mellitus, n=195** | 0.81 (0.28-2.33) | 0.69 | 1.07 (0.52-2.22) | 0.86 | 1.49 (0.64-3.48) | 0.35 |  |  |  |  |  |  |
| **Immunsuppressive treatment, n=170** | lim(OR)=0 | 0.02 | 0.63 (0.25-1.60) | 0.33 | 1.15 (0.44-3.02) | 0.77 |  |  |  |  |  |  |
| **Substance Abuse, n=116** | lim(OR)=0 | 0.10 | **3.28 (1.71-6.29)** | **0.0003** | 0.99 (0.30-3.29) | 0.99 |  |  | **2.76 (1.43-5.33)** | **0.01** |  |  |
| **Autoimmunity, n=106** | lim(OR)=0 | 0.13 | 0.62 (0.19-2.01) | 0.42 | **2.51 (1.01-6.20)** | **0.05** |  |  |  |  | **2.51 (1.01-6.20)** | **0.05** |
| **Liver disease, n=64** | lim(OR)=0 | 0.39 | 1.08 (0.33-3.55) | 0.90 | 2.69 (0.92-7.88) | 0.07 |  |  |  |  |  |  |
| **Renal disease, n= 63** | lim(OR)=0 | 0.40 | 0.71 (0.17-2.97) | 0.64 | 1.24 (0.29-5.29) | 0.77 |  |  |  |  |  |  |
| **Immune deficiency, n= 63** | lim(OR)=0 | 0.40 | 0.71 (0.17-2.97) | 0.64 | lim(OR)=0 | 0.36 |  |  |  |  |  |  |

**Additonal table 3.** Univariable and multivariable odds ratios (OR) with 95% confidence intervals (CI) for invasive pneumococcal disease with serotypes 7F, 9V and 14, included in PCV10, PCV13 and PPV23, in relation to patient derived factors in Southwest Sweden between 2009 and 2015. PCV10= 10-valent pneumococcal conjugate vaccine. PCV13= 13-valent pneumococcal conjugate vaccine. PPV23= 23-valent pneumococcal polysaccharide vaccine.

|  | **Odds ratio with 95 % confidence interval** | | | | | | | | | | |
| --- | --- | --- | --- | --- | --- | --- | --- | --- | --- | --- | --- |
| **Variable** | **Univariable analysis** | | | | | |  | **Multivariable analysis** | | | |
|  | **7F** (n=99) | ***p*** | **9V** (n=68) | ***p*** | **14** (n=67) | ***p*** | | **7F** (n=99) | ***p*** | **14** (n=67) | ***p*** |
| **Age (by 10 years)** | **0.82 (0.75-0.89)** | **<0.0001** | 0.91 (0.82-1.01) | 0.09 | 1.13 (0.99-1.30) | 0.08 | | **0.99 (0.98-1.00)** | **0.006** |  |  |
| **Men, n=668** | 1.38 (0.91-2.09) | 0.13 | 1.47 (0.90-2.43) | 0.13 | 0.76 (0.46-1.25) | 0.28 | |  |  |  |  |
| **Predisposing factor, any, n=1051** | **0.42 (0.27-0.66)** | **0.0001** | 0.92 (0.51-1.69) | 0.80 | 0.91 (0.49-1.66) | 0.75 | |  |  |  |  |
| **Smoking, n=528** | 1.19 (0.79-1.80) | 0.40 | 1.03 (0.63-1.69) | 0.91 | **0.52 (0.30-0.91)** | **0.02** | |  |  | **0.49 (0.28-0.86)** | **0.01** |
| **Cardiovascular disease, n=379** | **0.23 (0.11-0.45)** | **<0.0001** | 0.68 (0.38-1.22) | 0.19 | 1.48 (0.89-2.47) | 0.13 | | **0.28 (0.13-0.57)** | **0.0004** |  |  |
| **Malignancy, n=271** | 0.55 (0.30-1.01) | 0.05 | 0.57 (0.28-1.16) | 0.12 | 1.01 (0.55-1.84) | 0.98 | |  |  |  |  |
| **Pulmonary disease, n=267** | **0.51 (0.28-0.95)** | **0.04** | 1.67 (0.97-2.86) | 0.06 | 0.67 (0.34-1.33) | 0.25 | |  |  |  |  |
| **Diabetes mellitus, n=195** | 0.77 (0.41-1.44) | 0.41 | 0.54 (0.23-1.26) | 0.15 | 1.26 (0.66-2.39) | 0.49 | |  |  |  |  |
| **Immunsuppressive treatment,n=170** | **0.34 (0.13-0.84)** | **0.02** | **1.97 (1.09-3.59)** | 0.03 | 1.49 (0.78-2.84) | 0.23 | | **0.34 (0.14-0.86)** | **0.02** |  |  |
| **Substance Abuse, n=116** | 1.03 (0.50-2.09) | 0.94 | 0.80 (0.32-2.04) | 0.65 | 0.47 (0.14-1.51) | 0.20 | |  |  |  |  |
| **Autoimmunity, n=106** | 0.58 (0.23-1.46) | 0.25 | 1.55 (0.72-3.33) | 0.26 | **2.96 (1.56-5.62)** | **0.0009** | |  |  | **3.18 (1.66-6.07)** | **0.0005** |
| **Liver disease, n=64** | 0.19 (0.03-1.35) | 0.09 | 1.23 (0.43-3.48) | 0.70 | 0.58 (0.14-2.44) | 0.46 | |  |  |  |  |
| **Renal disease, n=63** | 0.39 (0.09-1.61) | 0.19 | 0.58 (0.14-2.44) | 0.46 | 1.27 (0.45-3.60) | 0.66 | |  |  |  |  |
| **Immune deficiency, n=63** | 0.60 (0.18-1.94) | 0.39 | 0.58 (0.14-2.44) | 0.46 | 0.59 (0.14-2.48) | 0.47 | |  |  |  |  |

**Additional table 4.** Univariable and multivariable odds ratios (OR) with 95% confidence intervals (CI) for invasive pneumococcal disease with PPV23 serotypes 8, 9N and 11A in relation to patient derived factors in Southwest Sweden between 2009 and 2015. PPV23= 23-valent pneumococcal polysaccharide vaccine. lim(OR)=0= No episodes of invasive pneumococcal disease.

|  | **Odds ratio with 95 % confidence interval** | | | | | | | | | | | |
| --- | --- | --- | --- | --- | --- | --- | --- | --- | --- | --- | --- | --- |
| **Variable** | **Univariable analysis** | | | | | | **Multivariable analysis** | | | | | |
|  | **8** (n=43) | ***p*** | **9N** (n=47) | ***p*** | **11A** (n=49) | ***p*** | **8** (n=43) | ***p*** | **9N** (n=47) | ***p*** | **11A** (n=49) | ***p*** |
| **Age (by 10 years)** | 0.95 (0.83-1.10) | 0.51 | 1.08 (0.93-1.27) | 0.31 | 1.03 (0.89-1.19) | 0.68 |  |  |  |  |  |  |
| **Men, n=668** | 1.33 (0.72-2.47) | 0.36 | 1.19 (0.66-2.13) | 0.57 | 0.70 (0.40-1.25) | 0.23 |  |  |  |  |  |  |
| **Predisposing factor, any, n=1051** | 1.05 (0.48-2.30) | 0.89 | 2.06 (0.81-5.27) | 0.13 | **2.78 (0.99-7.81)** | **0.05** |  |  |  |  |  |  |
| **Smoking, n=528** | **1.72 (0.94-3.17)** | **0.08** | 1.31 (0.73-2.34) | 0.37 | **2.01 (1.13-3.59)** | **0.02** |  |  |  |  |  |  |
| **Cardiovascular disease, n=379** | 0.83 (0.42-1.67) | 0.61 | 1.69 (0.93-3.07) | 0.08 | 1.08 (0.58-2.01) | 0.81 |  |  |  |  |  |  |
| **Malignancy, n=271** | **0.18 (0.04-0.75)** | **0.02** | 1.65 (0.87-3.13) | 0.12 | 1.55 (0.82-2.93) | 0.17 | **0.18 (0.04-0.75)** | **0.02** |  |  |  |  |
| **Pulmonary disease, n=267** | **2.15 (1.13-4.08)** | **0.02** | 0.79 (0.36-1.71) | 0.55 | 1.42 (0.74-2.72) | 0.29 | **2.16 (1.13-4.12)** | **0.02** |  |  |  |  |
| **Diabetes mellitus, n=195** | 0.74 (0.29-1.91) | 0.54 | 0.67 (0.26-1.71) | 0.40 | 1.29 (0.62-2.71) | 0.50 |  |  |  |  |  |  |
| **Immunsuppressive treatment, n=170** | 0.49 (0.15-1.61) | 0.24 | 1.61 (0.77-3.40) | 0.21 | 1.53 (0.73-3.21) | 0.26 |  |  |  |  |  |  |
| **Substance Abuse, n=116** | 1.36 (0.53-3.53) | 0.52 | 0.45 (0.11-1.86) | 0.27 | **2.78 (1.35-5.73)** | **0.006** |  |  |  |  | **2.78 (1.35-5.73)** | **0.006** |
| **Autoimmunity, n=106** | 0.54 (0.13-2.28) | 0.40 | **2.43 (1.10-5.34)** | **0.03** | 1.30 (0.50-3.35) | 0.59 |  |  | **2.40 (1.09-5.31)** | **0.03** |  |  |
| **Liver disease, n=64** | 0.94 (0.22-3.99) | 0.94 | 0.41 (0.06-3.04) | 0.38 | **2.88 (1.18-7.04)** | **0.02** |  |  |  |  |  |  |
| **Renal disease, n=63** | lim(OR)=0 | 0.23 | 0.87 (0.21-3.68) | 0.85 | 1.30 (0.39-4.30) | 0.67 |  |  |  |  |  |  |
| **Immune deficiency, n=63** | lim(OR)=0 | 0.23 | **3.75 (1.61-8.75)** | **0.002** | lim(OR)=0 | 0.17 |  |  | **3.72 (1.59-8.71)** | **0.003** |  |  |

**Additional table 5.** Univariable odds ratios (OR) with 95% confidence intervals (CI) for invasive pneumococcal disease with serotype 23F, included in PCV10, PCV13 and PPV23, 22F and 33F, included in PPV23 and the non-vaccine serotype 23A in relation to patient derived factors in Southwest Sweden between 2009 and 2015. PCV10= 10-valent pneumococcal conjugate vaccine. PCV13= 13-valent pneumococcal conjugate vaccine. PPV23= 23-valent pneumococcal polysaccharide vaccine. lim(OR)=0= No episodes of invasive pneumococcal disease.

|  | **Univariable odds ratio with 95 % confidence interval** | | | | | | | |
| --- | --- | --- | --- | --- | --- | --- | --- | --- |
| **Variable** | **22F** (n=135) | ***p*** | **23 A** (n=35) | ***p*** | **23 F (**n=47) | ***p*** | **33F** (n=59) | ***p*** |
| **Age (by 10 years)** | 1.03 (0.94-1.13) | 0.48 | 1.05 (0.88-1.24) | 0.61 | 1.03 (0.87-1.22) | 0.72 | 0.98 (0.87-1.11) | 0.79 |
| **Men, n=668** | 0.99 (0.70-1.42) | 0.98 | 0.80 (0.41-1.56) | 0.51 | 0.67 (0.34-1.32) | 0.25 | 0.74 (0.44-1.25) | 0.26 |
| **Predisposing factor, any, n=1051** | 1.19 (0.74-1.91) | 0.46 | 1.46 (0.56-3.79) | 0.44 | 1.00 (0.43-2.30) | 0.99 | 1.19 (0.59-2.38) | 0.63 |
| **Smoking, n=528** | 0.94 (0.66-1.36) | 0.76 | 0.50 (0.23-1.08) | 0.08 | 0.93 (0.47-1.84) | 0.84 | 0.74 (0.43-1.29) | 0.29 |
| **Cardiovascular disease, n=379** | 1.20 (0.82-1.76) | 0.34 | 0.98 (0.46-2.05) | 0.95 | 0.94 (0.45-1.96) | 0.86 | 0.83 (0.45-1.50) | 0.53 |
| **Malignancy, n=271** | 0.95 (0.61-1.48) | 0.81 | **2.63 (1.32-5.23)** | **0.006** | 0.92 (0.40-2.12) | 0.84 | 1.08 (0.58-2.03) | 0.81 |
| **Pulmonary disease, n=267** | 0.97 (0.62-1.51) | 0.89 | 0.80 (0.33-1.94) | 0.62 | 0.77 (0.32-1.87) | 0.57 | 0.89 (0.45-1.73) | 0.72 |
| **Diabetes mellitus, n=195** | 0.80 (0.47-1.37) | 0.42 | 0.73 (0.25-2.09) | 0.56 | 1.94 (0.90-4.19) | 0.09 | 1.65 (0.87-3.12) | 0.12 |
| **Immunsuppressive treatment, n=170** | 0.75 (0.42-1.34) | 0.33 | 1.12 (0.43-2.91) | 0.82 | 1.35 (0.55-3.29) | 0.51 | 0.75 (0.32-1.76) | 0.51 |
| **Substance Abuse, n=116** | 0.45 (0.19-1.04) | 0.06 | lim(OR)=0 | 0.07 | 0.60 (0.14-2.51) | 0.48 | 0.74 (0.26-2.07) | 0.56 |
| **Autoimmunity, n=106** | 0.79 (0.39-1.60) | 0.51 | 1.93 (0.73-5.08) | 0.18 | 0.32 (0.04-2.33) | 0.26 | 1.30 (0.54-3.09) | 0.56 |
| **Liver disease, n=64** | 0.41 (0.13-1.33) | 0.14 | 1.18 (0.28-5.03) | 0.82 | lim(OR)=0 | 0.32 | lim(OR)=0 | 0.10 |
| **Renal disease, n=63** | **2.14 (1.11-4.12)** | **0.02** | 1.20 (0.28-5.12) | 0.81 | 0.56 (0.07-4.12) | 0.57 | 1.46 (0.51-4.17) | 0.48 |
| **Immune deficiency, n=63** | 1.47 (0.71-3.06) | 0.30 | 1.20 (0.28-5.12) | 0.81 | 0.56 (0.07-4.12) | 0.57 | 1.06 (0.32-3.48) | 0.93 |
